# Potential interactive effect of positive expectancy violation and sleep on memory consolidation in dogs

**DOI:** 10.1038/s41598-024-60166-8

**Published:** 2024-04-25

**Authors:** Vivien Reicher, Tímea Kovács, Barbara Csibra, Márta Gácsi

**Affiliations:** 1 Clinical and Developmental Neuropsychology Research Group, HUN-REN Research Centre for Natural Sciences, Budapest, Hungary; 2 Department of Ethology, Eötvös Loránd University, Budapest, Hungary; 3 Doctoral School of Biology, ELTE Eötvös Loránd University, Budapest, Hungary; 4 HUN-REN–ELTE Comparative Ethology Research Group, Budapest, Hungary

**Keywords:** Learning and memory, Reward, Animal behaviour

## Abstract

In dogs, as in humans, both emotional and learning pretreatment affect subsequent behaviour and sleep. Although learning often occurs in an emotional-social context, the emotion-learning interplay in such context remain mainly unknown. Aims were to assess the effects of Controlling versus Permissive (emotional factors) training (learning factors) styles on dogs’ behaviour, learning performance, and sleep. Family dogs (*N* = 24) participated in two command learning sessions employing the two training styles with each session followed by assessment of learning performance, a 2-h-long non-invasive sleep EEG measurement, and a retest of learning performance. Pre- to post-sleep improvement in learning performance was evident in dogs that received the Permissive training during the second learning session, indicating that dogs that experienced a more rewarding situation than expected (positive expectancy violation) during the second training session showed improved learning success after their afternoon sleep. These results possibly indicate an interactive effect of expectancy violation and sleep on enhancing learning.

## Introduction

It is a common experience that emotional events are more memorable than non-emotional ones, and these emotional memories often remain vivid for years. Human and rodent experimental data confirm that the occurrence of an emotional event during a learning period can enhance memory consolidation and synaptic plasticity^1,2^ and that emotionally salient, especially negative–relative to neutral–stimuli are often prioritized for consolidation^3,4^. The enhancing effects of emotional experiences on long-term memory are also reflected in differential activity of brain regions implicated in emotional and memory processes (amygdala and medial temporal lobes), both during memory encoding and sleep^5,6^.

Human literature shows that sleep also preferentially strengthens memories with emotional valance^7,8^, with data showing selective consolidation of both negative^9–11^ and positive^12^ materials. The majority of available research linked the consolidation of emotional memories to rapid eye movement (REM) sleep (e.g.^10,13,14^), although some recent work linked consolidation also to non-rapid eye movement (NREM) sleep^11,15^. For example, selective consolidation of negative information is positively associated with the amount of REM sleep and REM theta power activity^10,16^ as well as with NREM sleep and NREM delta power activity^17^.

Memory consolidation also seems to be protected by sleep when individuals learn new yet competing information (interference effect). Proactive interference occurs when later learned information is attenuated by earlier learned information and retroactive interference occurs when earlier learned information is attenuated by later learned information^18^. Sleep has been observed to reduce the interference effect in both humans^19,20^ and in non-human animals, i.e., songbirds^21^.

Although a substantial portion of learning, especially in children, takes place within socio-emotional contexts (e.g., at home with parents, in school with peers and teachers), the available knowledge on the interaction between emotion and learning is primarily based on adult human and rodent data obtained in laboratory studies where learning occurred in a laboratory environment and alone, without social context^22^. Accordingly, the current understanding of the interplay between emotion and learning may not be generalizable to everyday situations where learning occurs within a socio-emotional context. Here, socio-emotional context indicates emotionally positive (permissive; both social and food reinforcement in case of correct action and no scolding in case of incorrect action) and emotionally negative/stressful (controlling; food but no social reinforcement in case of correct action and soft scolding in case of incorrect action) training styles. The aim of this study was to gain an understanding of the interplay between emotion and learning that is generalizable to such real-world situations. To achieve this aim, the family dog seemed to be an ideal subject, as dog training always occurs within a socio-emotional context involving a dog trainer or owner. Further, data indicate that the family dog (*Canis familiaris*) is a reliable and valid animal model to study complex human functions^23^ not only at the behavioural but also at the neural level (for review, see^24,25^).

Training methods^26^, training styles^27,28^, duration and frequency of training sessions^29^, and the type of activity after learning^30,31^ have all been reported to influence the dogs’ memory performance. Consistent with the human and rodent literature^32–34^, in dogs, mild stress enhances^35,36^ whereas strong stress impairs learning^28^. Further, both pro- and retroactive interference effects were also apparent in the case of dogs, particularly in the context of olfaction^37^ and spatial learning^38^.

Regarding sleep, data suggest notable comparability across dog and human sleep^24^. Employing a non-invasive canine polysomnography method, the dog natural sleep architecture was described^39–42^ and the associations between awake functioning and sleep were examined^43,44^. Earlier findings showed that both emotional pretreatment^45^ and learning^31^ affect dog sleep macrostructure and EEG spectra. In a previous study a negative (owner separation, approach by threatening stranger) and a positive (ball play and petting) pretreatment was applied before sleep and the negative treatment was associated with decreased drowsiness and increased REM sleep^45^. Regarding learning, Kis and colleagues found evidence of sleep-dependent memory consolidation in dogs and they also found that post-sleep performance improvement showed a negative association with REM delta and a positive association with REM beta power activity^31^. Yet the interactive effect of emotional pretreatment and learning still needs to be examined both on behavioural and neural levels.

To address the identified gap in knowledge about the interplay between emotion and learning in socio-emotional, real-world situations, in the current study, family dogs participated in two command learning sessions that differed with regard to the socio-emotional context of learning.

After an adaptation sleep recording, dogs were trained to associate new commands with known actions (e.g., give paw, lie down) during two subsequent command learning sessions (on different days), with the controlling and permissive conditions presented in a balanced within-subject design. Both learning sessions were followed by an assessment of learning performance and a 2-h-long afternoon sleep EEG measurement. After sleep, learning performance was retested to assess any changes in performance and to examine how these changes related to the sleep EEG characteristics. Primary aim was to examine the main and interactive effects of (i) training style, (ii) order/ interference effect, and (iii) sleep on learning performance in dogs. To validate the condition manipulation, during trainings the inner state of the dogs was evaluated. Stress in dogs can be assessed using different methods (e.g.^35,46–48^), including behavioural observation targeting contact-seeking with the owner^49^. Studies on dogs’ attachment behaviour provided evidence for human-analogue safe haven effect of the owner in a potentially dangerous situation, where owners can provide a buffer against stress in dogs^50^. The fact that dogs show a tendency to get in the proximity of the owner in a moderately stressful environment confirms the effective use of this behavioural variable to assess stress. Thus, behavioural data (time spent in close proximity to the owner) was used as a proxy of the inner state of the dog. Based on the literature, we hypothesized three potential outcomes regarding dogs' contact-seeking behaviour and learning performance, depending on the level of stress induced by the controlling training style; i) if it evokes considerable stress, it is likely to result in heightened contact-seeking behaviour towards the owner during training, as well as poorer learning performance during the test and retest, in comparison to the permissive training style; ii) if it elicits only a marginal level of stress, it may still result in higher contact-seeking behaviour in dogs, but their learning performance may be relatively better than that of the permissive training style; iii) if it does not elicit stress in dogs, there may be no discernible difference in contact-seeking behaviour or learning performance between the two training styles. Considering sleep parameters, previous research has demonstrated the effects of emotional pretreatment^45^ and learning^31^ on dog sleep variables. However, the interactive effect of these two factors has not been explored. Thus, we refrained from formulating hypotheses regarding the specific direction of change in sleep variables. Yet, as previous studies showed sleep-dependent memory consolidation in dogs^31^, we hypothesized that dogs will show better learning performance (performance improvement) after sleep.

## Methods

### Ethics statement

All experimental protocols were approved by the Hungarian “Scientific Ethics Committee for Animal Experimentation” (PE/EA/853–2/2016; PE/EA/00035-4/2023) and were conducted in accordance with Hungarian regulations on animal experimentation and Guidelines for use of animals in research, as outlined by the Association for the Study Animal Behaviour (ASAB). Prior to participation, owners received detailed information about the aims, circumstances, and features of the experiments and signed an informed consent form. All methods were carried out in accordance with relevant guidelines and regulations, and were approved by the United Ethical Review Committee for Research in Psychology (EPKEB; Ref. no.: 2023-04).

### Subjects

Owners participated on a voluntary basis and were recruited through the Department of Ethology participant pool and website, popular social networking sites, and via snowball sampling. Prior to participation, owners completed an online questionnaire assessing the demographics and sleep habits of their dog (e.g., *How often does the dog sleep in a familiar environment in the presence of the owner (who is engaged in other activities; meeting, work) during the afternoon*^42^*?*), training experience and the owner’s training style (e.g., *To what extent do you use play, praise, food reward or leash tug, hand correction and shout on a 0–100% scale?*). Owners who utilized leash tug, hand correction, and scolding for at least 30% of their interactions were classified as strict, while those who used these methods less frequently were categorized as soft. Training experience and owner training style were balanced in our sample. Furthermore, we considered the level of motivation, exclusively including dogs that displayed a preference for food, as indicated in the questionnaire responses.

*N* = 27 family dogs were recruited. One of them did not fulfil the criterion in the baseline test (see *Adaptation* section), and two were excluded from analyses due to excessively long training sessions (76.22 and 100 min long sessions, while the rest of the duration of sessions was between 18.42 and 55.22 min; mean duration of sessions was *M* = 35.14, *SD* = 10.03 min). Due to technical reasons, data loss occurred (three video recordings and one sleep EEG recording were not saved), thus behavioural data were evaluated in *N* = 21 dogs, learning performance data in *N* = 24, and sleep data in *N* = 23 dogs.

The 24 dogs whose data were used for analyses were 1–9 years old (*M*age = 4.35, *SD* = 2.24); 11 males and 13 females (10 male and 9 female dogs had been neutered); from 11 different breeds and 6 mutts. In between-group comparisons, dogs were age-matched.

### Experimental setup

Training sessions, tests and retests were conducted in a behaviour laboratory equipped with a chair and a camera system. During the experiment, the owner was sitting behind the dog trainer/experimenter and, to ensure that the dog remains focused on the designated task and the owner do not influence the dog behaviour, the owner was asked to be engaged with their cell phone and listen to music on headphones.

During the training sessions (occasions 2 and 3), a cage filled with meat and toys was placed in the laboratory (See Supplementary Fig. S1). This cage served to lure and distract the dogs, allowing the dog trainer to demonstrate a more pronounced difference between training styles while focusing the dog’s attention to the task. The training sessions started/continued if the dog sat and looked at the dog trainer (DT) from 1.5 m from the distractor.

Sleep measurements were conducted in a sleep laboratory fully equipped for non-invasive canine EEG measurements. A mattress and a reading lamp for the comfort of the owner and the dog provided a calm, dark and quiet environment for the dog to settle and fall asleep, while the experimenter controlled the data acquisition from outside of the laboratory (see details in^42^).

### General procedure

Each dog participated on three occasions on three different days, each approximately one week apart (see Table 1). The aim of occasion 1 was twofold; first, it served to familiarise the dog with the experimenter and with attachment of electrodes for the EEG measurement as well as to familiarise the dog with the sleep laboratory to attenuate or prevent a first-night effect—a phenomenon evidenced in both dogs^42^ and humans^51^, manifesting as marked macrostructural differences between the first and second sleep occasions in the laboratory. Second, to conduct a behavioural pretest as a prerequisite for further participation. Based on Hungarian commands and hand signals, dogs had to reliably perform the following four tasks from a sitting position: “lie down”, “give paw”, “turn around”, and “stand up”. This was important because in the first part of the training sessions, dogs had to learn to perform these actions for the already known hand signals and new English commands.

**Table 1:** Summary of the steps of all three occasions.

Occasion 1 (Adaptation + pretest)	Occasion 2 and 3
24-trial baseline test (Hungarian commands and hand gestures)	Training session (two actions per occasion, in four blocks of min 10 trials*) *(permissive/controlling training style, in counterbalanced order)*
	18-trial test session (English commands) *(neutral style)*
Sleep adaptation—EEG recording (1.5 h—not analysed)	Sleep EEG recording (2 h)
	18-trial retest session (English commands) *(neutral style)*

*Additional trials were conducted if the dog did not learn the task.

**Figure 1:**
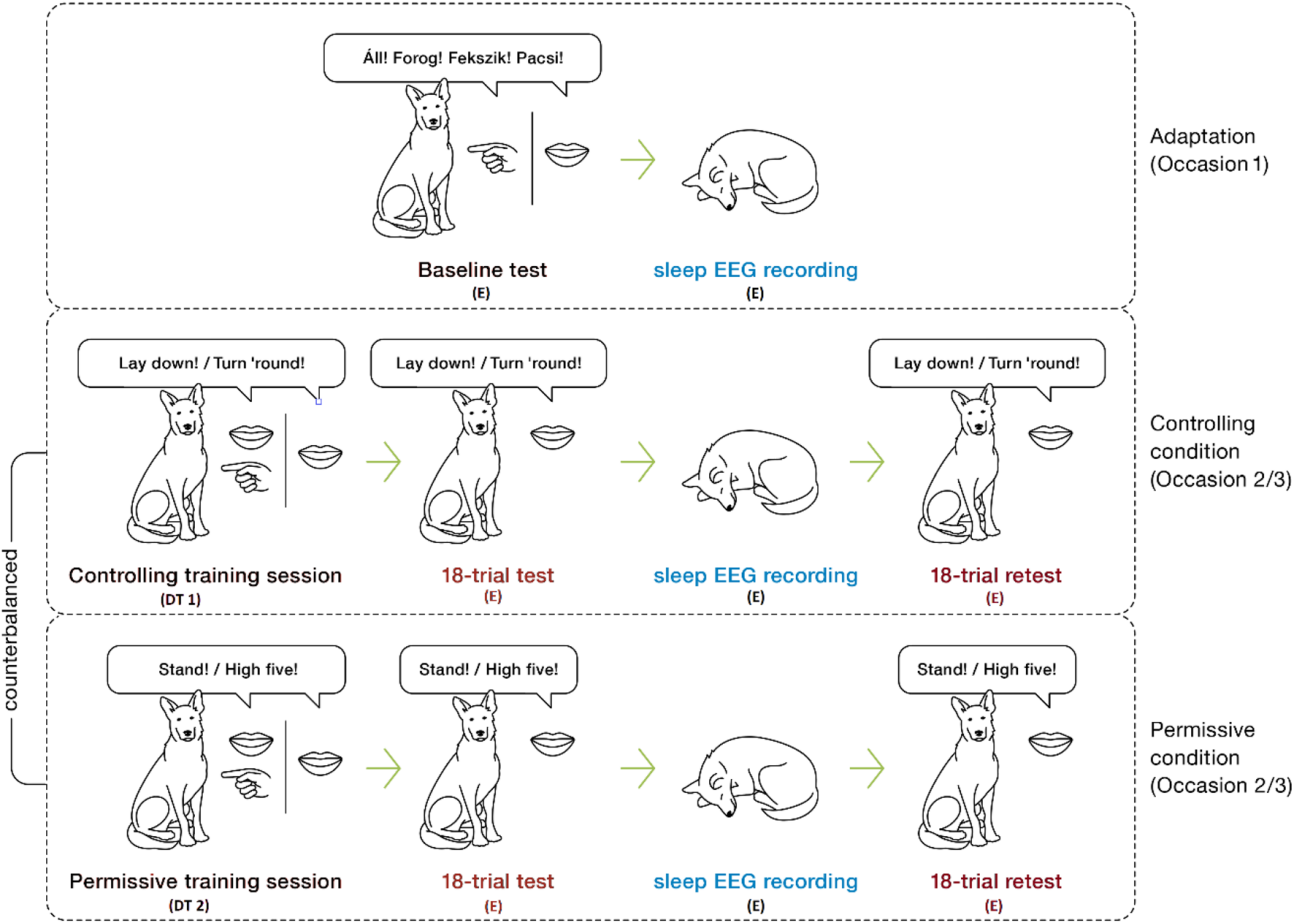
The structure of the learning paradigm. All dogs first attended an adaptation session and sleep data were collected for 1.5 h. Subsequently, the controlling and permissive conditions followed in counterbalanced order, each followed by another two-hour-long sleep recording session. Hand signals were only used during the baseline test and the trainings; only verbal commands were given in the test and retest. DT 1: dog trainer 1 and 2, E: experimenter. Illustrations adapted from^64^. Please note that the commands presented in the figure are illustrative examples, and all four commands were equally counterbalanced between conditions and occasions.

Both occasion 2 and 3 had four parts: a training session, a test session, a sleep EEG measurement, and a retest session (see Fig. 1). During the training sessions, the dog was required to learn to associate two out of the four known actions (one trick and one position, e.g., turn around, lie down/give paw, stand up) with new commands (English commands instead of the familiar Hungarian ones). For example, dogs had to learn to lie down to “lay down” (new command) instead of “fekszik” (old command). A different set of two out of the four known actions was used during occasion 2 and 3 (i.e., a different set of two actions for each of the two training styles). None of the dogs had any previous experience with the English commands used in the study.

During the training sessions, two different training styles were applied: controlling and permissive. Tests following the training sessions were performed in a neutral style (detailed description in *Training styles* section). The training procedure was followed by an 18-trial test session where the dog had to perform the actions for the previously learnt English commands (test). The test sessions were followed by a 2-h-long non-invasive sleep EEG measurement. After sleep, the learning performance was retested in another 18-trial test session (retest).

The experiment required three participants: an experimenter (E) and two dog trainers (DT 1 and DT 2). E conducted the baseline test and adaptation sleep on occasion 1, and the test, retest, and sleep EEG measurements on occasion 2 and 3. E was blind to the experimental manipulations. DT 1 and DT 2 trained the dog on occasion 2 and 3, respectively. The role of DT 1 and DT 2 was fulfilled by three experienced trainers (both in dog training and ethological experiments), all females and of similar ages (29 and 35). Prior to the study, pilot experiments were conducted to standardise the behaviour of DTs in the two conditions. Both the condition and occasion were counterbalanced between the dog trainers.

## Detailed Procedure

The structure of the learning paradigm is summarised in Fig. 1.

### Adaptation

To confirm that dogs reliably perform the four tasks, their performance was tested in a 24-trial baseline test, and continued participation was allowed if dogs performed the correct action in at least 75% of the trials. One dog was excluded from further parts of the experiment due to not reaching this criterion. In the second part of the training sessions, actions were requested only for the new English commands (for details see *Training session* section). The baseline test was followed by a 1.5-h-long adaptation sleep EEG measurement. All sleep adaptation recordings were successful.

### Training session

#### English commands

Occasion 2 and 3 started with training where the dog had to learn to associate known actions (lie down, turn around, give paw, stand up) with new commands (instead of the familiar, Hungarian commands, the unfamiliar, English commands). The familiar, Hungarian commands were matched with known English commands in number of syllables. Thus, the used commands were: (1) “Lay down!” for lie down, (2) “Turn ‘round!” for turn-around, (3) “High five!” for give paw, (4) “Stand!” for stand up. The duration of the training sessions was 18.42–55.22 min long, performed in four blocks separated by short breaks.

#### Structure

The basic structure of the training sessions was based on a previous protocol by Kis and colleagues^31^, with minor modifications. In the current study, the dog was requested to learn to associate two known actions with two unfamiliar commands, as indicated by the ability to perform the known actions to the new command from a sitting position. In each training session, the requested actions were a body position (lie down or stand up) and a trick (give paw or turn around).

The training sessions always followed the same pattern and contained four blocks; in block 1, the dog had to execute the first action (e.g., turn around) following the English command and the known hand signal. In block 2, the dog had to execute the first action following the English command without the hand signal. In both blocks, if the dog properly executed the action ten times, the session continued. The training of the second action (e.g., stand up) in block 3 and 4 followed the same structure. The blocks were separated by 5–10-min breaks.

#### Test and retest

After the training sessions with DTs, the owner was requested to walk outside with the dog for 10–15 min. The break was followed by an 18-trial test with E, where the dog had to execute the two actions for the newly learned English commands without hand signals; a total of 18 trials of “Turn ‘round!” and “High Five!” or “Lay down!” / “Stand!” in a fixed pseudorandom order (e.g., for “Turn ‘round!” and “Stand!”: TSTSTSSTTSTSSTTSTS). E was always neutral in style; when the dog performed the action correctly, she gave the dog a treat and praised the dog (e.g., “Good girl!”) using a neutral voice, but gave no further positive reinforcement. If the dog performed incorrectly, E moved away and called the dog (so that the dog followed E) and presented the next command. The test was immediately followed by a 2-h-long EEG sleep measurement (for details, see *Polysomnography* section). After sleep, learning performance was retested by E with the same 18-trial test session (retest). The mean duration of the tests and retests was *M* = 7.3 min (*SD* = 3.3), and *M* = 6.3 min (*SD* = 2.6), respectively (calculated on *n* = 37 and *n* = 38 due to missing video recordings).

### Training styles

During training sessions (on occasions 2 and 3), training styles were distinguished on the basis of three main aspects; DTs behaved differently when the dog (1) correctly performed the requested action correctly, (2) did not perform the requested action or incorrectly perform the requested action, and (3) approached the distractor cage.

In the controlling condition, in case of (1), DT rewarded the dog with a treat but did not praise the dog (used neither verbal nor physical reinforcement). In case of (2), DT scolded the dog (“No!”, “Not good!”, “What are you doing?!”) using a firm but not threatening voice, then moved away (so that the dog also moved with her) and repeated the same command. In case (3), DT called the dog firmly and used inhibiting commands (“No!”, “Leave it!”).

In the permissive condition, in case of (1), DT rewarded the dog with a treat and praised the dog using a combination of verbal and physical reinforcement (stroking, petting), depending on the dog’s preference. In case of (2), DT moved away, called the dog with a high-pitched voice (until the dog followed her) and encouraged the dog, then repeated the same command. In case of (3), DT called the dog kindly and tried to lure the dog away without using any inhibiting commands.

### Polysomnography

**Figure 2:**
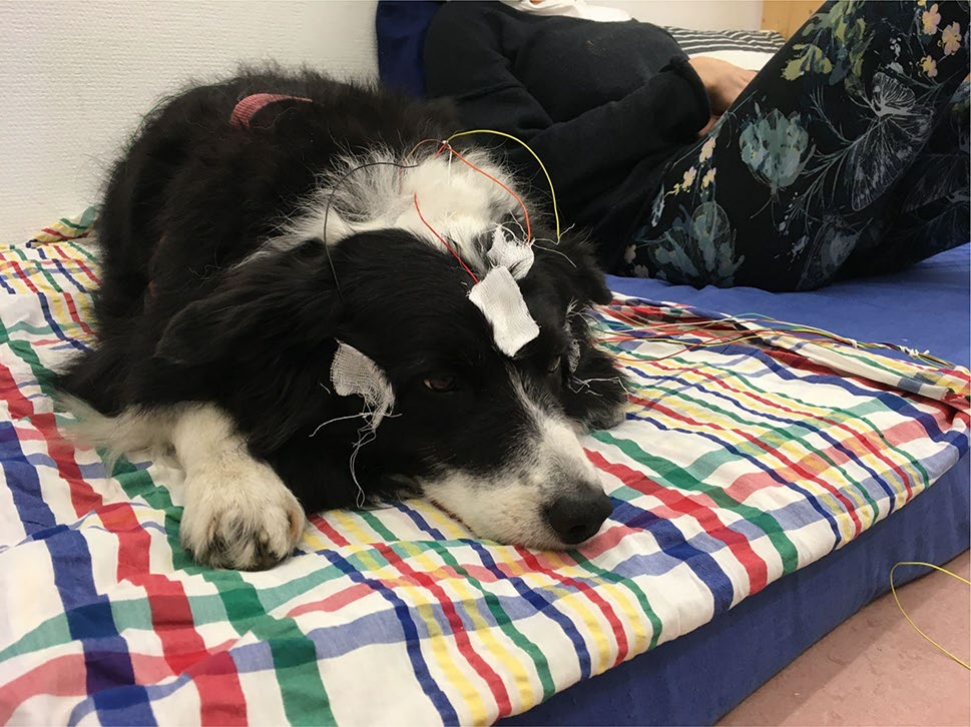
Electrode placement. Photo of a dog with electrodes in the presence of the owner, right before the onset of the sleep measurement.

Sleep was monitored by polysomnography (PSG) which allowed the parallel recording of electroencephalogram (EEG), electrooculogram (EOG), electrocardiogram (ECG) and respiration (PNG). In this research, we followed the previously validated PSG method on dogs^41^ with the single addition of another electrode on the right zygomatic arch, allowing us to detect four EEG channels and an eye movement channel^42^. With this new setup instead of two, four EEG channels, and an eye movement channel had been recorded. The two electrodes placed on the right and left zygomatic arch next to the eyes (F8, F7) and the scalp electrodes over the anteroposterior midline of the skull (Fz, Cz) were referred to the G2, a reference electrode which was in the posterior midline of the skull (occiput; external occipital protuberance). The ground electrode (G1) was attached to the left musculus temporalis. See Fig. 2 for a photo of a dog with electrode placement and a detailed drawing of a dog with the names and exact placement of the electrodes (Supplementary Fig. S2). For the recordings, gold-coated Ag/AgCl electrodes were used, secured by Signa Spray Electrode Solution (Parker, United States) and EC2 Grass Electrode Cream (Grass Technologies, United States).

The analysed recordings in our study were obtained with one of the following two technical arrangements:

1. In the case of 19 recordings the signal was collected, amplified and digitised at a sampling rate of 1000 Hz/channel, using the 40-channels NuAmps amplifier (© 2018 Compumedics Neuroscan) with applying DC-recording, and saved in .cnt format with the Scan 4.3 Acquire software (© 2018 Compumedics Neuroscan).
2. In the case of 27 recordings the signal was collected, pre-filtered, amplified and digitised with a sampling rate of 1024 Hz/channel using a SAM 25 R style MicroMed Headbox (MicroMed Inc., Houston, TX, USA). The hardware passband was set at 0.5–256 Hz, sampling rate of 512 Hz, anti-aliasing filter with cut-off frequency at 1 kHz, and 12-bit resolution covering a voltage range of ± 2 mV as well as second-order software filters (high pass > 0.016 Hz, low pass < 70 Hz) using System Plus Evolution software (MicroMed Inc, Houston, TX, USA).

To overcome differences in EEG filter characteristics between EEG recording devices, a calibration process was applied. For details, see^52^.

## Data analysis

Behaviour and learning performance were coded in Solomon Coder based on video recordings. Learning performance data were also live-coded by E on a paper, thus the loss of video recordings did not affect these data. Finally, performance was calculated as the percent of correct actions in both test and retest.

During training sessions, the duration of the dog being in close proximity to the owner was coded in seconds and calculated to percentages. Originally, we examined a range of behaviours, such as yawning, scratching and vocalisation (e.g., whining). However, these examined behaviours occurred with such low frequency that the corresponding data were not analysable. As food reward was provided to the dogs, lip licking was excluded as a behaviour to be coded, as its occurrence could potentially be misleading. Proximity seeking towards the owner occurred often enough to be used for analyses, thus we found it as the only reliable and analysable indicator of stress in the dogs.

Sleep recordings were visually scored by two experienced researchers (VR and TK) in accordance with standard criteria^53^, adapted for dogs^41^. For inter-rater reliability, the two researchers scored 4 dog measurements (540 epochs/recording). Reliability was very high (Cohen’s κ = 0.73). A self-developed program (by Ferenc Gombos; Fercio’s EEG Plus, 2009—2023) was used to analyse and export data. The recordings were manually scored, and the program provided data for exporting macrostructural variables. This manual coding reliably identifies the stages of wake, drowsiness, NREM and REM in dogs^54^. For figure illustrating the different sleep stages, see Supplementary Fig. S3. It also needs to be noted that in case of dog sleep, different stages of NREM are not distinguished, but handled uniformly as SWS or NREM sleep (for details see^41^). For statistical analysis the three macrostructural variables of interest were: Sleep efficiency (the percentage of time spent asleep during the sleep measurement. Relative NREM duration (calculated as the percentage of time spent in NREM) and relative REM duration (calculated as the percentage of time spent in REM).

Regarding spectral power analysis, we calculated and extracted NREM delta power activity for the Fz channel which provided the most artefact-free traces. Dogs that had no NREM (*n* = 3) were excluded. As in 13 recordings, dogs did not reach REM sleep, REM spectral power analyses were not conducted. Artefact rejection of the EEG trace was carried out manually in 4 s epochs. Average power spectral values (1–30 Hz) were calculated by a mixed-radix Fast Fourier Transformation (FFT) algorithm, applied to the 50% overlapping, Hanning-tapered 4 s windows of the EEG signals of the Fz-G2 derivation. The relative power spectra were calculated as the proportion of total power (1–30 Hz) and frequency ranges of NREM delta (1–4 Hz).

All variables are summarised in Supplementary Table S1.

### Statistical analysis

Data were analysed with generalized linear mixed models (GLMM) with IBM SPSS Statistics, version 26. Before conducting our analysis, we performed the Shapiro–Wilk normality test on our variables. The results indicated that the data deviated from normality (all showing positive skewed distributions) in case of being in close proximity to the owner, REM sleep, and NREM delta power activity (*p* < 0.05). Consequently, to mitigate the positive skewness in our dataset, we applied various normalization transformations, such as square roots, logarithms, and reciprocal transformations. Despite these attempts, the skewness persisted in our data. Therefore, in case of these variables, models were fitted using the Gamma distribution. Other variables such as learning performance (test, retest, and performance improvement), sleep efficiency, NREM sleep showed a Gaussian distribution. In our models, learning performance and sleep variables were entered as targets, whereas condition (permissive, controlling), order of conditions (permissive first, controlling second (*n* = 9) or controlling first, permissive second (*n* = 12) and their interaction (order of conditions*condition) were included as fixed factors. Subject ID was included as a random effect to account for inter-individual differences. The best fitting model was identified separately by backward selection using the Akaike Information Criterion (AIC).

## Results

### Behaviour: being in close proximity to the owner

**Figure 3:**
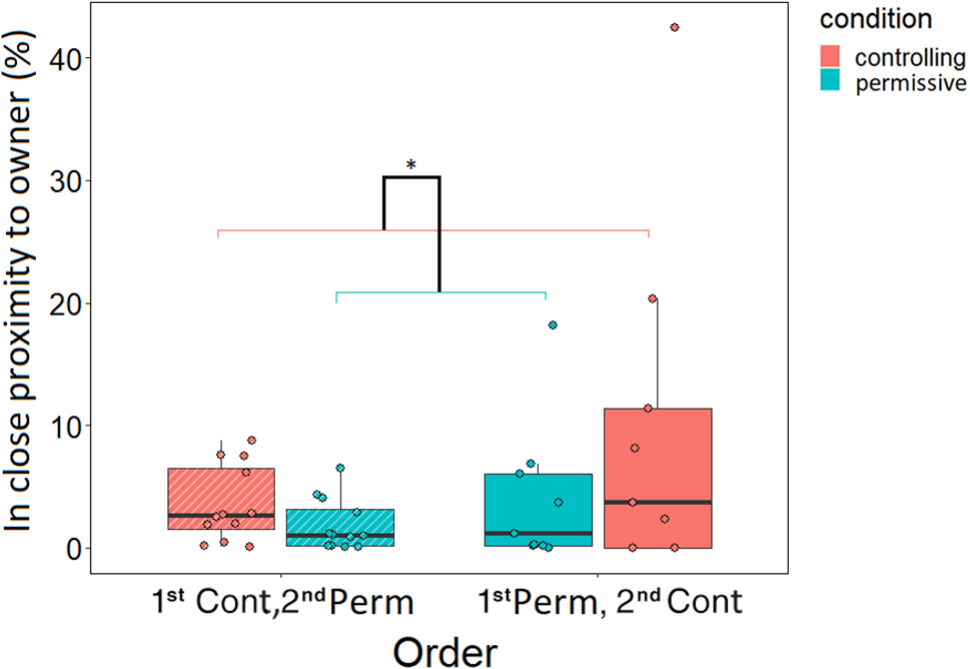
The effect of condition on the relative time spent in close proximity to the owner. *p < 0.05. The percentage value was calculated by dividing the minutes spent in close proximity to the owner by the total duration of the learning session and then multiplying it by 100. The diagonal pattern indicates a subsample of dogs (n = 12) that had a controlling training on their first session and a permissive training on the second. The “plain” bars indicate the other subsample of dogs (n = 9) that had a permissive training on their first session and a controlling training on the second.

Regarding the relative time spent in close proximity to the owner, order had no main effect (F_1,38_ = 0.581 *p* = 0.451) but condition did (F_1,38_ = 6.723 *p* = 0.013): dogs spent more time in close proximity to their owner during the controlling condition, compared to the permissive condition (Fig. 3). The interaction of order*condition was not significant (F_1,38_ = 1.976, p = 0.168).

### Learning performance

#### Test and retest performances

**Figure 4:**
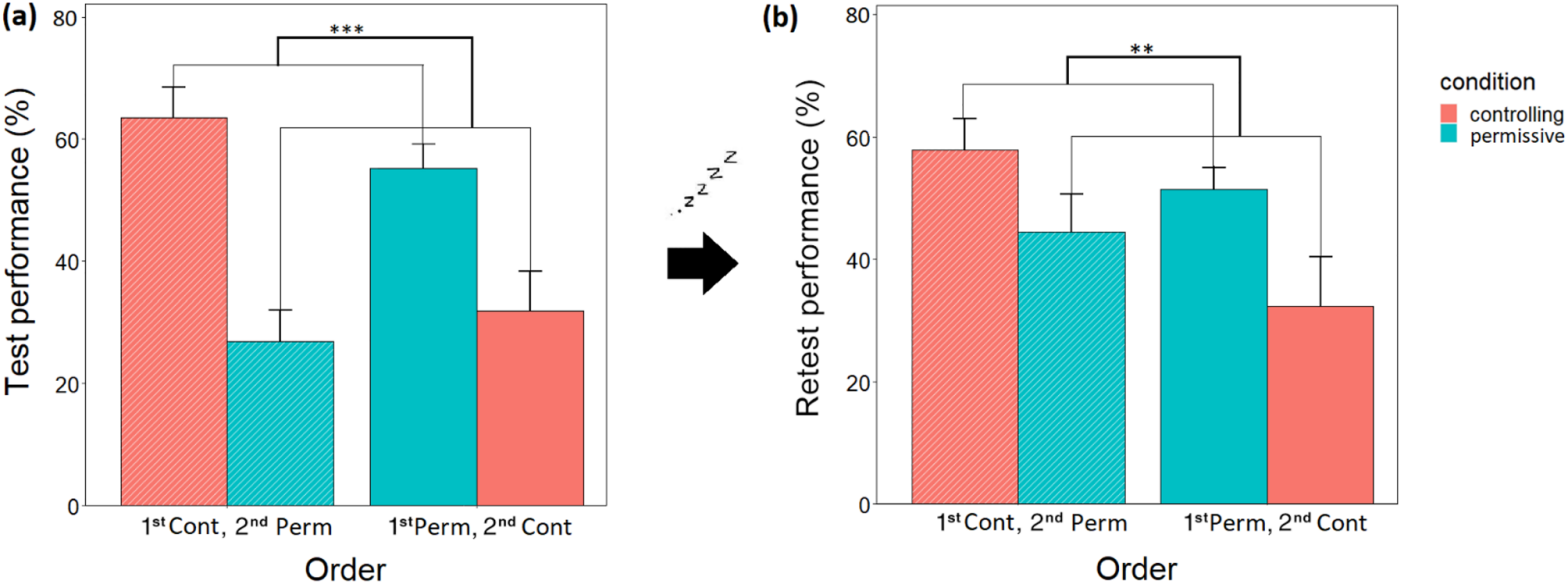
The effect of order on (**a**) test (mean +/− SE) and (**b**) retest (mean +/− SE) performance. **p < 0.01, ***p < 0.001. The percentage values were calculated by dividing the number of correct actions by the total requested actions and then multiplying it by 100. The diagonal pattern indicates a subsample of dogs (n = 12) that had a controlling training on their first session and a permissive training on the second. The “plain” bars indicate the other subsample of dogs (n = 12) that had a permissive training on their first session and a controlling training on the second.

Condition did not affect test (F_1,44_ = 1.589, *p* = 0.214) and retest (F_1,44_ = 0.232, *p* = 0.63) performance, but order had a main effect on both test (F_1,44_ = 32.446, *p* < 0.001) and retest (F_1,44_ = 7.568, *p* = 0.009) performance. The interaction of condition and order did not have an effect on test (F_1,44_ = 0.131, *p* = 0.719) and retest (F_1,44_ = 2.81, *p* = 0.101) performance. Dogs performed better on occasion 2 regardless of the condition, compared to occasion 3 (Fig. 4).

#### *Performance improvement (*= *retest-test performance)*

**Figure 5:**
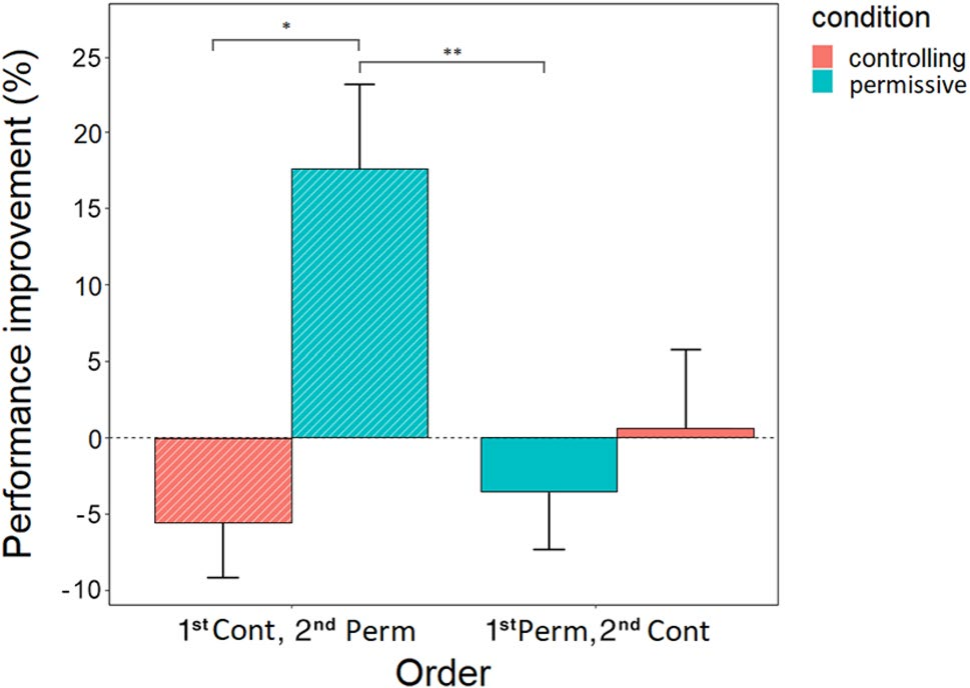
Order-specific effect of condition on performance improvement (mean + /- SE). Performance improvement (= retest-test performance) occurred in case of those dogs that had a permissive condition on occasion 3. *p < 0.05; **p < 0.01. The percentage value was calculated by subtracting the retest performance percentage from the test performance percentage. The diagonal pattern indicates a subsample of dogs (n = 12) that had a controlling training on their first session and a permissive training on the second. The “plain” bars indicate the other subsample of dogs (n = 12) that had a permissive training on their first session and a controlling training on the second.

Condition had an order-specific effect on performance improvement (F_1,44_ = 4.68, *p* = 0.036). Post hoc comparisons revealed that dogs that had permissive condition on occasion 3 had greater performance improvement, compared to their controlling condition on occasion 2 (*p* = 0.002 [8.241;34.072]). Similarly, dogs that had permissive condition on occasion 3 had greater performance improvement, compared to dogs that had a permissive condition on occasion 2 (*p* = 0.024 [2.377;31.535]) (Fig. 5).

Since performance improvement occurred in one subsample (dogs that had permissive condition on their second training session), we further tested whether the degree of performance improvement correlated with NREM, REM sleep and NREM delta power activity in each subsample. The performance improvement was not related to NREM, REM sleep, and NREM delta power activity (all* p* > 0.05). Note that REM spectral data were not analysed due to insufficient artifact-free REM traces in *n* = 5 dogs, further reducing the sample size.

#### Incorrect actions during test and retest

To test the interference hypothesis, the types of incorrect actions during tests and retests on occasion 3 were further analysed. The type of incorrect actions were: (i) learnt on the previous occasion, (ii) learnt on the same occasion, (iii) no action performed, (iv) different action: any performed action that was not one of the four tasks included in the study. In case of test performance, more incorrect actions occurred because dogs incorrectly executed the actions learnt on the same occasion (learnt same occasion → different action: *p* < 0.001 [18.732;70.657]; learnt same occasion → no action: *p* = 0.011 [− 21.658;30.93]) and on the previous occasion (learnt previous occasion → different action: *p* < 0.001 [16.69;63.428]; previous occasion → no action: *p* = 0.016 [4.0761;63.428]). No differences were detected between the incorrect actions learnt on the same and previous occasion (*p* = 0.730 [− 21.658;30.93]). For the proportion of incorrect actions during tests, see Supplementary Fig. S4.

Similarly, in case of retest performance, more incorrect actions occurred because dogs incorrectly executed the actions learnt on the same occasion (learnt on the same occasion → different action: *p* = 0.001 [12.729;72.266]; learnt on the same occasion → no action: *p* = 0.017 [4.415;63.762]) and on the previous occasion (learnt previous occasion → different action *p* = 0.002 [9.449;60.419]; learnt previous occasion → no action: *p* = 0.0037 [1.218;51.832]). No differences were detected between the incorrect actions learnt on the same and previous occasion (*p* = 0.611 [− 21.580;36.707]). For the proportion of incorrect actions during retest, see Supplementary Fig. S3.

### Sleep

#### Sleep efficiency

**Figure 6:**
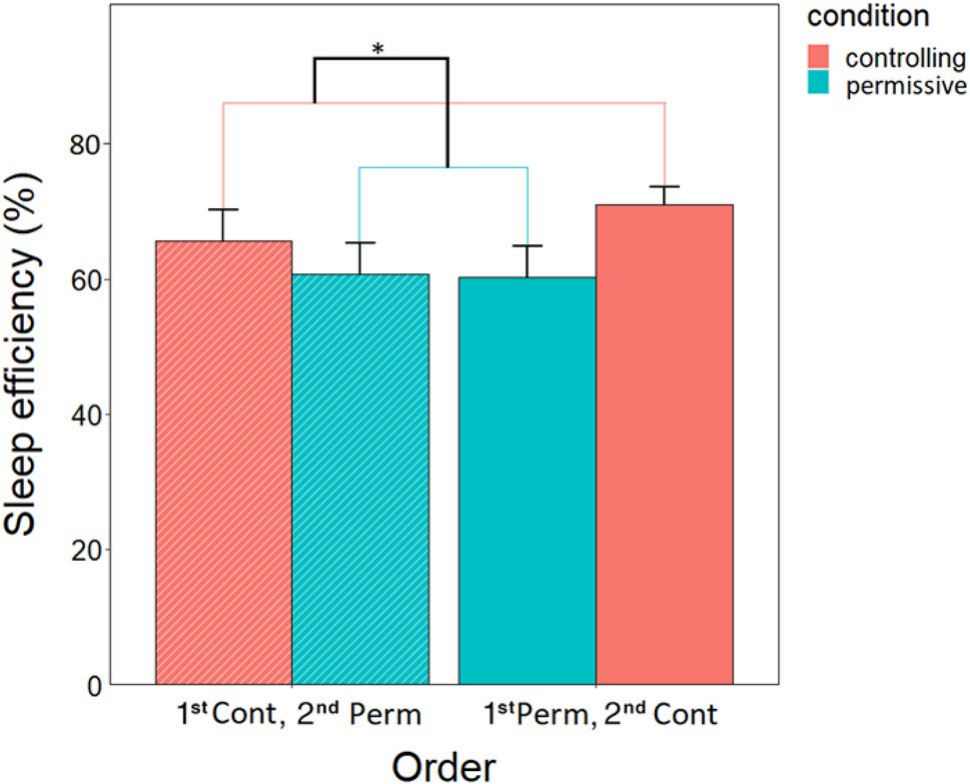
Effect of condition on Sleep efficiency. *p < 0.05. The percentage value was calculated by dividing the minutes spent sleeping by the total duration of sleep recording and then multiplying it by 100. The diagonal pattern indicates a subsample of dogs (n = 11) that had a controlling training on their first session and a permissive training on the second. The “plain” bars indicate the other subsample of dogs (n = 12) that had a permissive training on their first session and a controlling training on the second.

Condition had a main effect on sleep efficiency (F_1,42_ = 5.812, *p* = 0.020), specifically, dogs slept more after the controlling condition, compared to the permissive condition (Fig. 6). Order (F_1,42_ = 1.045, *p* = 0.313) and the interaction of order and condition (F_1,42_ = 0.199, *p* = 0.658) had no effect on sleep efficiency.

#### Relative duration of NREM and REM sleep

Condition, order and their interaction did not influence relative NREM duration (condition: F_1,42_ = 1.672, *p* = 0.203; order: F_1,42_ = 2.527, *p* = 0.119) and REM sleep (condition: F_1,42_ = 0.085, *p* = 0.772; order: χ^2^ F_1,42_ = 0.161, *p* = 0.690). The interaction of condition*order had no effect on NREM sleep (F_1,42_ = 0.006, p = 0.939) but showed a trend-level effect on REM sleep (F_1,42_ = 3.562, *p* = 0.066). Post-hoc comparisons revealed that dogs that had a controlling condition on occasion 3 spent more time in REM sleep compared to those dogs that had the controlling condition first, on occasion 2 (p = 0.059).

#### NREM delta power activity

Condition (F_1,40_ = 0.172, *p* = 0.680), order (F_1,40_ = 0.183, *p* = 0.671) and the interaction of order*condition (F_1,40_ = 0.040, *p* = 0.842) did not affect NREM delta power activity.

## Discussion

Using a repeated command learning paradigm, our goal was to investigate the main and interactive effects of training style, learning and sleep in the family dog. We report associations between training style and behaviour as well as the interaction effect of training style and its order on learning performance and sleep macrostructure.

First, to determine if the different training styles elicited differences in the inner state, we assessed proximity-seeking behaviour towards the passive owner as an indicator of stress^49^. In line with our hypothesis, even though the controlling training session did not involve punishment and/or threat, dogs spent more time in close proximity to their owner during the controlling training session, compared to the permissive one, suggesting that in dogs, controlling training style elicits more stress.

Despite extensive evidence on negative emotional memories and stress being selectively consolidated^3,4^, and on further selective consolidation of those by sleep^9–11^, in the current study, no such effect was observed. We did not see performance improvement after the controlling trainings during the pre-sleep test and post-sleep retest. Further, even after the first permissive training session, we did not observe any improvement in learning performance. This lack of improvement could potentially be attributed to the likelihood that this training might have induced mild stress in dogs, stemming from that both the environment and the trainer were unfamiliar to them and/or the lack of attention from/interaction with the owner. It seems that, contrary to some findings^35^, even mild stress can impair dogs’ learning success in some learning context. To further explore this, additional research is warranted under conditions where no stress is applied.

Although training style alone did not, the order of sessions did influence learning performance during the pre-sleep test and post-sleep retest. Specifically, regardless of condition, dogs exhibited better learning performance in the test and retest following the first training session (occasion 2) compared to the test and retest that followed the second training session (occasion 3). This aligns with previous research documenting proactive interference during an olfactory learning task, whereby previous learning hindered the ability to acquire new information in the same learning context^37^. However, retroactive interference during a spatial learning task has also been reported^38^, whereby learning new information impaired the memory of previously learned information. In combination with the current findings, this suggests that the type of interference might depend on the type of memory task. To date, we haven’t found a study that examined the interference effect in a declarative memory task (e.g., command learning) in dogs. It is noteworthy that unlike what is apparent in humans^19,20^, sleep did not reduce the impact of interference, as we observed a strong main effect of order even during the post-sleep retest.

Regarding pre- to post-sleep improvement in learning performance, we observed a strong interactive effect of condition and order. Only those dogs exhibited improvements in performance from the first training session to the second for whom the controlling session preceded the permissive session. This finding might reflect expectation violation or prediction error; both occur when there is a discrepancy between the events that are expected based on prior experiences and the events that are actually encountered^55^. Both aversion-^56,57^ and reward-^58–60^ related prediction error promote memory consolidation. Although the learning paradigm in the current study was not specifically designed to manipulate or probe expectation violation or prediction error, it is reasonable to posit that some form of those took place and results seemed to be consistent with this hypothesis. Specifically, when in the second training session, the actual event was more pleasant (and thus rewarding) than what was expected based on the prior experience of the first training session, improved performance was apparent. However, when in the second session, the actual event was less pleasant than expected, no improvements in performance were observed. This effect was evident only after a 2-h sleep, possibly indicating an interactive effect of expectancy violation and sleep on enhancing learning. Our results indicate a central role of positive reinforcement both in enhancing their performance and welfare. In future research it would be worthwhile to examine how subsequent permissive training sessions affect the dogs’ performance, elucidating whether the observed effect stems from the positive expectation violation or the positive training per se. Furthermore, it's important to note that we did not include a wake condition in our study to directly assess whether sleep/rest played a role as this was not the primary focus of our investigation. Implementing a wake control would be crucial for a more accurate comprehension of the impact of sleep. While Kis and colleagues have previously found evidence of sleep-dependent memory consolidation in dogs using a similar learning design, their study did not examine the interplay between emotional factors and learning; it focused solely on the learning aspect^31^. Therefore, incorporating a wake condition in future research could provide a clearer understanding of the true influence of sleep in such emotional-learning conditions in dogs.

No direct associations were found between improved performance and characteristics of sleep (macrostructural or spectral). This finding is consistent with recent sleep spindle analysis performed on a sample that partially overlapped with the current sample^61^. Although there is evidence for an association between memory consolidation and sleep in both humans^10,62,63^ and dogs^31,51^, these results are not always replicated^64–66^. Contradictory findings emphasize the complexity of the relation between memory processes and sleep, and call for more standardized experimental methods^67^. The current study most closely aligns with earlier dog sleep EEG studies, where rather similar procedures were used^31,51^. However, in the current study, dogs faced a more difficult task; they had to learn twice as many new commands, learned across both conditions, and had a 2-h-long afternoon sleep. In combination with the combined effect of emotional and learning pretreatment, this more complex design may have negatively affected their success, as also indicated by the evident interference effect. Additionally, Kis, Szakadát and colleagues found an association between learning performance and REM delta and beta power activity^31^. In the current sample, most dogs did not have enough REM sleep or artefact-free REM sleep to analyse, so the association between learning and REM could not be tested yet it is possible that in dogs, sleep-dependent memory consolidation is better reflected in REM sleep. Despite the absence of a direct association between learning and sleep, it was evident in some (a subsample of) dogs that sleep promoted learning, indicated by the pre- to post-sleep improvement in learning performance.

In terms of the effect of training styles (condition) on sleep, macrostructural but not spectral differences were observed. Dogs slept more after the controlling than after the permissive session. There was also an order-specific trend-level condition effect on REM sleep; dogs that had a controlling condition on occasion 3 spent more time in REM sleep compared to dogs that had a controlling condition on occasion 2. Contrary to hypotheses, there was no condition effect on NREM sleep. In a previous study, a negative (owner separation, approach by threatening stranger) and a positive (ball play and petting) pretreatment was applied before sleep and although no differences in sleep efficiency were observed, the negative treatment was associated with decreased drowsiness and increased REM sleep^45^. These partially congruent findings may be attributed to methodological differences, as Kis and colleagues examined the effects of socio-emotional pretreatment alone, whereas we investigated the combined effect of socio-emotional pretreatment and learning. Our results suggest that in dogs, sleep may have an important role in emotion processing and memory consolidation that transpire through intertwined processes.

Considering the complex nature of this study, our results may have been influenced by factors not accounted for in analyses. For instance, differences in dog personality traits, level of motivation, owners’ style of training, or the dogs’ prior experiences in training and working with unfamiliar trainers may have contributed to variance in the data. However, in dog research these cofounding factors usually contribute to mild variance^28,67,68^, to attenuate most of the effect of these unaccounted factors, dogs were carefully selected and balanced with permissive and strict owners and training levels within conditions.

Dogs, being popular companion animals, live in close proximity with humans. Consequently, finding the “golden” training style or training method for dogs can be regarded as a hot topic in dog behavioural studies, although this may also depend on the particular individual and the context. This is also intriguing if we take into account the growing body of research suggesting that family dogs exhibit behaviours resembling to human disorders, such as autism spectrum disorder (ASD)^69^ or attention-deficit/hyperactivity disorder (ADHD)^69,70^. As dogs naturally show notable ADHD-related phenotypic variability^71,72^ and gene polymorphisms^73^, it would be interesting to examine how dogs with ADHD-like traits (e.g., with inattention and/or impulsivity problems) react in similar, interdependent training situations or how expectancy violation would affect their learning success. Finding suitable training methods for these dogs would improve their quality of life.

## Conclusion

Our knowledge on interactions between emotion, learning and sleep is primarily based on data obtained in laboratory studies were learning occurs in a non-social context. Accordingly, our aim with this study was to investigate the interplay between emotion and learning in subsequent test situations that may be generalizable to everyday situations, using dogs as subjects.

Our results indicated that regardless of condition, learning performance was better during the first training session, suggesting that dogs’ success during the second session was influenced by proactive interference. Pre- to post-sleep improvement in learning performance was observable only on the second training session, possibly due to the interactive effect of sleep and expectancy violation. Dogs that experienced a more rewarding situation than expected during the second training session, showed improved learning success after their afternoon sleep. However, pre- to post-sleep improvement in learning performance was not associated with sleep macrostructure and spectra, but the training condition did affect sleep macrostructure. However, to accurately assess the interactive effect of expectancy violation and sleep, further research is necessary. This may involve incorporating additional components, such as subsequent permissive training sessions to see whether expectancy violation caused the learning improvement and not the permissive training itself. Additionally, including a wake condition would allow for a direct assessment of the role of sleep in this context.

These outcomes hold substantial significance as they offer potential generalizability to everyday situations with high ecological validity. Our results indicate a central role of positive reinforcement both in enhancing their performance and welfare. Of import, soft scolding and/or the lack of social reinforcement seemed to be enough to elicit stress-related behaviour (proximity seeking towards the owner) in dogs, it is crucial to pay close attention to their training techniques. Especially since some family dogs with serious behavioural problems may differ in their sensitivity and responsiveness to the various training techniques, which is an important aspect from an animal welfare point of view. Furthermore, future research holds the potential to expand the investigation to human participants, employing a similarly more generalizable research design, thus allowing for a more comprehensive exploration of the parallels between dogs and humans in this regard.

### Supplementary Information

Supplementary Information.

## Data Availability

The datasets generated during and/or analysed during the current study are available from the corresponding author on reasonable request.
