# Supplementary material for: Potential interactive effect of positive expectancy violation and sleep on memory consolidation in dogs
Source: Sci Rep. 2024 Apr 25;14:9487. doi: 10.1038/s41598-024-60166-8 (PMC11045790; doi:10.1038/s41598-024-60166-8)
Supplement: Supplementary file 1 — Supplementary Information. [file 41598_2024_60166_MOESM1_ESM.docx]

**Potential interactive effect of positive expectancy violation and sleep on memory consolidation in dogs**

**Vivien Reicher**^1,2,3,^***, Tímea Kovács**^2,3^**, Barbara Csibra**^2,3^**, and Márta Gácsi**^2,4^

^1^Clinical and Developmental Neuropsychology Research Group, Institute of Cognitive Neuroscience and Psychology, Research Centre for Natural Sciences, Budapest, Hungary

^2^Department of Ethology, Eötvös Loránd University, Budapest, Hungary

^3^Doctoral School of Biology, ELTE Eötvös Loránd University, Budapest, Hungary

^4^ELKH-ELTE Comparative Ethology Research Group, Budapest, Hungary

*vivien.reicher@gmail.com


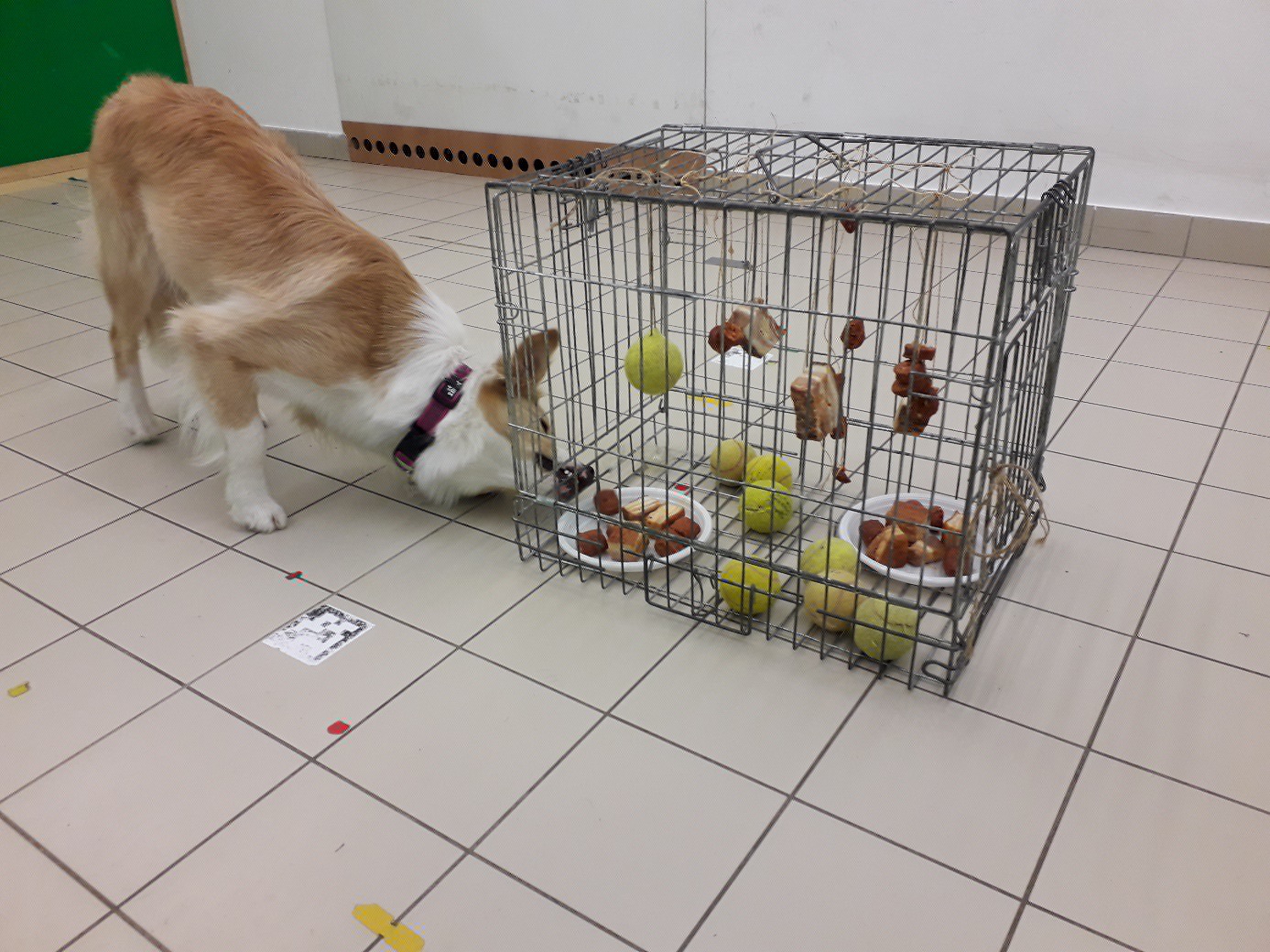


SFig. S1: Distractor cage. During the training sessions, a cage filled with meat and toys was placed in the laboratory to lure and distract the dog from learning.


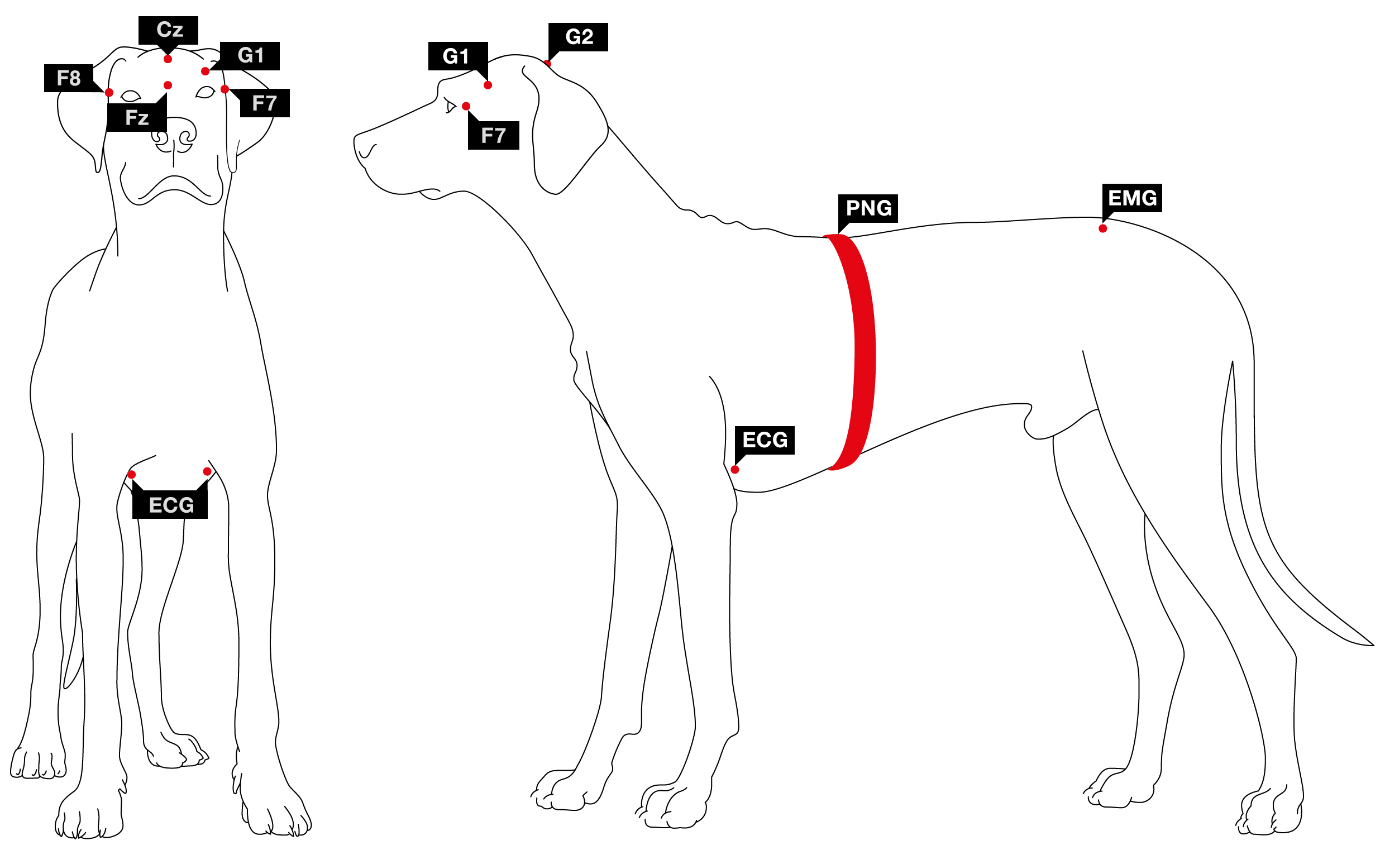


SFig. S2: Electrode placement. Placement of the electrodes and the respiratory belt (Fz-Cz: frontal and central midline; F7-F8: left and right electrodes placed on the zygomatic arch; G2: reference electrode; G1: ground electrode; ECG: electrocardiographic electrodes; EMG: electromyography electrodes; PNG: respiratory belt − respiratory inductance plethysmography).


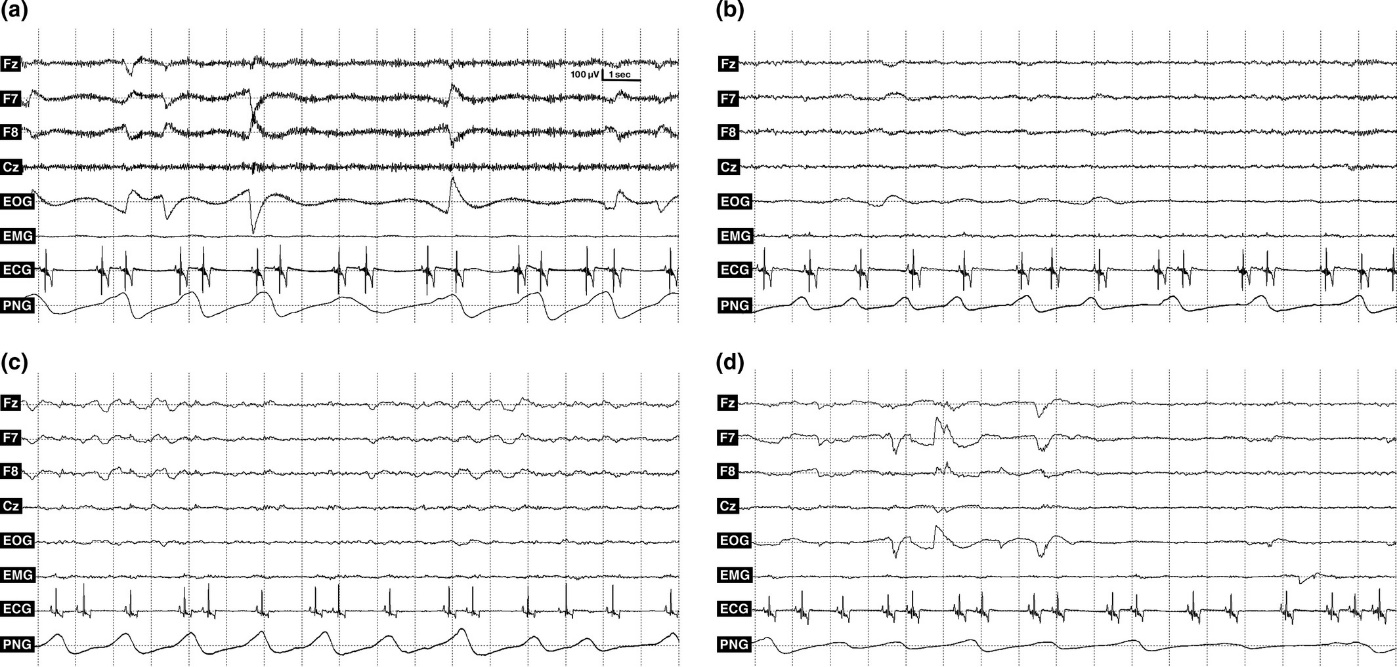


SFig. S3: Representative polysomnographic (PSG) traces from the sleep stages of (a) wake, (b) drowsiness, (c) non-rapid eye movement (NREM), (d) rapid eye movement (REM).


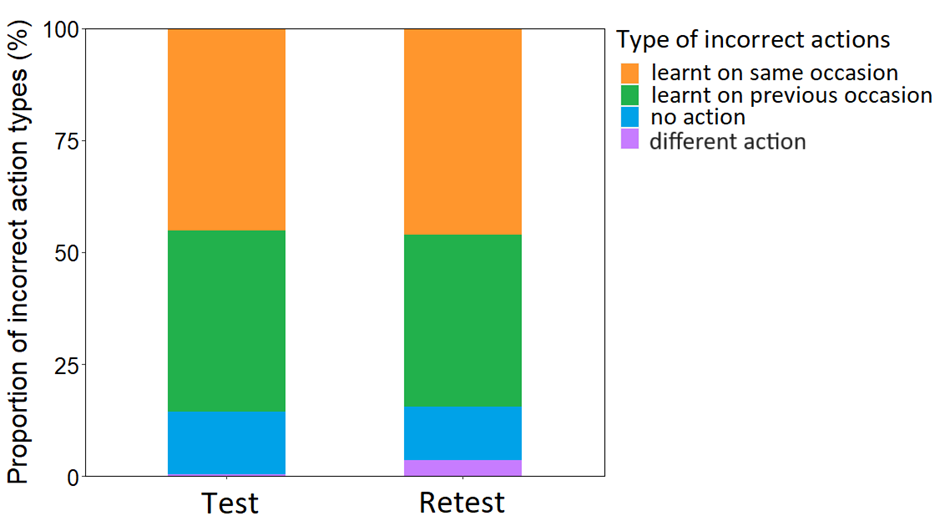


SFig. S4: Incorrect action on occasion 3. Proportion of incorrect actions during test and retest of occasion 3. The type of incorrect actions were: i) learnt on the previous occasion, ii) learnt on the same occasion, iii) no action performed, iv) different action: any performed action that was not one of the four tasks included in the study.

| **Phase** | **Variable** | **Definition** |
| --- | --- | --- |
| Training | Time spent in close proximity to the owner | Percentage of time spent within 30 cm radius of the owner |
| Test and retest | Performance | Percentage of correct actions |
|  | Performance improvement | Subtraction of test performance from retest performance |
|  | Incorrect actions* | Types of incorrect actions: i) learnt on the previous occasion, ii) learnt on the same occasion, iii) no action performed, iv) different action: any performed action that was not one of the four tasks included in the study |
| Sleep** | Sleep efficiency | Percentage of time spent asleep during the sleep measurement |
|  | Relative NREM duration | Percentage of time spent in NREM |
|  | Relative REM duration | Percentage of time spent in REM |
|  | NREM delta power activity | The proportion of total power (1–30  Hz) and frequency ranges of NREM delta (1–4 Hz) |

STable S1: Coded variables and their definition in the phases of the experiment. *Incorrect actions were only coded on occasion 3 to test the proactive interference hypothesis.** Drowsiness sleep stage was coded but not analysed.
